# Tyrosine kinase chromosomal translocations mediate distinct and overlapping gene regulation events

**DOI:** 10.1186/1471-2407-11-528

**Published:** 2011-12-28

**Authors:** Hani Kim, Lisa C Gillis, Jordan D Jarvis, Stuart Yang, Kai Huang, Sandy Der, Dwayne L Barber

**Affiliations:** 1 Campbell Family Cancer Research Institute, Ontario Cancer Institute, University Health Network, 610 University Avenue, Toronto, Ontario, M5G 2M9, Canada; 2 Department of Medical Biophysics, Faculty of Medicine, University of Toronto, Toronto, Ontario, Canada; 3 Department of Laboratory Medicine and Pathobiology, Faculty of Medicine, University of Toronto, Toronto, Ontario, Canada

## Abstract

**Background:**

Leukemia is a heterogeneous disease commonly associated with recurrent chromosomal translocations that involve tyrosine kinases including BCR-ABL, TEL-PDGFRB and TEL-JAK2. Most studies on the activated tyrosine kinases have focused on proximal signaling events, but little is known about gene transcription regulated by these fusions.

**Methods:**

Oligonucleotide microarray was performed to compare mRNA changes attributable to BCR-ABL, TEL-PDGFRB and TEL-JAK2 after 1 week of activation of each fusion in Ba/F3 cell lines. Imatinib was used to control the activation of BCR-ABL and TEL-PDGFRB, and TEL-JAK2-mediated gene expression was examined 1 week after Ba/F3-TEL-JAK2 cells were switched to factor-independent conditions.

**Results:**

Microarray analysis revealed between 800 to 2000 genes induced or suppressed by two-fold or greater by each tyrosine kinase, with a subset of these genes commonly induced or suppressed among the three fusions. Validation by Quantitative PCR confirmed that eight genes (Dok2, Mrvi1, Isg20, Id1, gp49b, Cxcl10, Scinderin, and collagen Vα1(Col5a1)) displayed an overlapping regulation among the three tested fusion proteins. Stat1 and Gbp1 were induced uniquely by TEL-PDGFRB.

**Conclusions:**

Our results suggest that BCR-ABL, TEL-PDGFRB and TEL-JAK2 regulate distinct and overlapping gene transcription profiles. Many of the genes identified are known to be involved in processes associated with leukemogenesis, including cell migration, proliferation and differentiation. This study offers the basis for further work that could lead to an understanding of the specificity of diseases caused by these three chromosomal translocations.

## Background

Chromosomal translocations are the most frequently occurring genetic abnormalities in leukemias and they exploit a mechanism by which normal regulatory pathways are subverted, thereby providing a proliferative advantage to a leukemic clone. Many of the chromosomal translocations involve tyrosine kinases. In most cases, translocations juxtapose a tyrosine kinase domain to another protein containing an oligomerization motif. For example, BCR-ABL is generated by t(9;22)(q34;q11) [1], which fuses the N-terminus of BCR to the C-terminus of ABL. TEL-PDGFRB (t(5;12)(q33;p13)) [2] and TEL-JAK2 (t(9;12)(p24;p13)) [3,4] fuse the N-terminus of TEL to the C-terminus of PDGFRB or JAK2, respectively. In all three fusions, the N-terminal translocation partners contain an oligomerization domain (i.e. the coiled-coil domain of BCR or the pointed domain of TEL). The oligomerization domain mediates ligand-independent activation of the kinase domains, causing factor-independence *in vitro *and leukemia-like diseases *in vivo*.

The kinase fusions provide a proliferative signal for the leukemic clone to expand, while additional mutations are necessary to fully elicit the leukemia phenotype. For all three fusions, the enhanced proliferative capacity is associated with activation of STAT proteins [5-13], various members of MAP kinases [14-18] and activation of the PI3K pathway [19-22] (reviewed in [23-25]). While BCR-ABL, TEL-PDGFRB and TEL-JAK2 activate overlapping signaling pathways, the relative contribution and requirement for these pathways downstream of each fusion is not clear. Given that each of these pathways can transmit signals to alter gene transcription, it is possible that their signals converge at the level of gene transcription, resulting in a similar gene expression pattern. Alternatively, they may result in distinct gene expression patterns depending on the relative significance of different pathways downstream of the three fusions.

The three fusions give rise to distinct diseases according to the WHO classification (reviewed in [26]). BCR-ABL is predominantly described in patients with chronic myelogenous leukemia (CML). The TEL-PDGFRB translocation is commonly associated with chronic myelomonocytic leukemia (CMML), a disease characterized by a preferential expansion of monocytes [26,27]. TEL-JAK2 has been described in three distinct diseases: pre-B cell acute lymphoblastic leukemia (ALL), T-cell ALL and atypical CML. The differences in the clinical features of the three fusion tyrosine kinases may be, in part, due to distinct regulation of gene transcription.

In order to investigate this possibility in an unbiased manner, we used microarray analysis to compare the effects of BCR-ABL, TEL-PDGFRB and TEL-JAK2 on gene expression in Ba/F3 cells. We demonstrated that BCR-ABL, TEL-PDGFRB and TEL-JAK2 elicit distinct gene expression changes. Notably, TEL-PDGFRB, but not the other two fusions, induces the expression of Stat1 and Gbp1. Interestingly, Cd55 and Ndrg1 are induced by BCR-ABL and suppressed by TEL-PDGFRB, while Cxcl10 and Scinderin are commonly regulated by all three fusions. This study illustrates that oncogenic tyrosine kinases can trigger distinct gene regulation, which may contribute to specificity downstream of each fusion protein.

## Methods

### Cell lines and culture

Ba/F3 cells and Ba/F3 TEL-JAK2(5-19) cells were cultured as previously described [10,16,20]. TEL-JAK2(5-19) was introduced via electroporation and individual subclones were isolated by limiting dilution.

Ba/F3 BCR-ABL cells were generated by retroviral infection of Ba/F3 cells with pMSCV BCR-ABL p210 construct. Ba/F3 TEL-PDGFRB cells were generated by electroporation. Both cell lines were maintained in RPMI-complete media supplemented with murine IL-3, Geneticin and 100 ng/ml of Imatinib. Individual G418-resistant subclones were isolated by limiting dilution, and expression of BCR-ABL and TEL-PDGFRB was confirmed by immunoprecipitation and immunoblot analysis.

### Cell cycle analysis

2 × 10^6 ^cells were collected, washed once in cold PBS, fixed in ice-cold 70% ethanol and stored at -20°C. For propidium iodide staining, cells were washed twice in cold PBS and incubated in PBS containing RNAse A (Invitrogen) (1 unit per 10^6 ^cells) for 30 min at 37°C. After 30 min, 50 μg/ml of propidium iodide (Roche Boehringer Mannheim, Laval, QC) was added to the cell suspension, and cells were analyzed by FACScan. The cell cycle profile was determined by using the ModFit LT^® ^(Verity Software House, Topsham, ME).

### Imatinib dose-response assay

Ba/F3 cells stably expressing pMSCV TEL-PDGFRB or pMIGR BCR-ABL were washed three times in 10 mM HEPES (pH 7.4)/Hanks balanced salts, and incubated in RPMI-complete medium for 24 h at 37°C. After 24 h, 2 × 10^5 ^cells were resuspended in RPMI-complete media containing specific amounts of Imatinib mesylate dissolved in DMSO. Ba/F3 cells were resuspended in RPMI-complete media containing 100 pg/ml of IL-3 and increasing amounts of Imatinib. As a vehicle control, Ba/F3 were resuspended in RPMI-complete media containing DMSO only.

### RNA extraction

Ba/F3 cells were washed three times in 10 mM HEPES (pH 7.4)/Hanks balanced salts, incubated in RPMI-complete medium for 5 h at 37°C and stimulated with 2 ng/ml of rmIL-3 for 0, 8, 12, 24 h and 1 week at 37°C.

Ba/F3 BCR-ABL and Ba/F3 TEL-PDGFRB cells were washed three times and incubated in RPMI-complete media and Imatinib (1 μg/ml for Ba/F3 BCR-ABL cells and 100 ng/ml for Ba/F3 TEL-PDGFRB cells) for 5 h at 37°C. After 5 h, cells were washed three times and incubated in RPMI-complete media for 0, 8, 12, 24 h and 1 week at 37°C.

Ba/F3 TEL-JAK2 cells were washed three times and incubated in RPMI-complete media for 0, 8, 12, 24 h and 1 week at 37°C.

For all cell lines, cells were harvested at each time-point for total RNA extraction, which was performed by using QIAGEN RNAeasy MINI^® ^(Qiagen, Mississauga, Ontario, Canada), and the integrity of the RNA samples was assessed by running a formaldehyde denaturing gel according to the manufacturer's protocol.

### Oligonucleotide array experiment and data analysis

Further RNA quality assessment, sample RNA *in vitro *transcription, labeling and hybridization were conducted by the Toronto Centre for Applied Genomics at the Sick Children's Hospital http://www.tcag.ca/ according to the standard Affymetrix protocols. Hybridization was performed using the Affymetrix mouse MOE430A oligonucleotide array, which represents 13247 unique mouse gene transcripts. All arrays have been scaled to a target intensity of 150, and data was analyzed by using Affymetrix Micro Array Suite (MAS) 5.0 software to calculate expression values for each transcript and Detection p-values. The Detection *p*-value was calculated by the Detection algorithm, which assigned a Present (*p *< 0.04), Marginal (0.04 <*p *< 0.06) or Absent (0.06 <*p*) call, and reflected the confidence of the detection of the given transcript. All of the reported induced genes displayed the Detection score of Present or Marginal at the 1 week point, and all of the reported suppressed genes displayed the Detection score of Present or Marginal at the 0 h point. Ratios between the expression levels at the 0 h and all subsequent time-points were calculated.

For gene expression in Ba/F3 TEL-JAK2 cells, expression values obtained from Ba/F3 TEL-JAK2 cells at the 1 week time point were compared to the expression values obtained from Ba/F3 cells at the 0 h time point.

### Clustering analysis

Gene expression values were log2 transformed, and two hundred and fifty of the most highly induced genes and two hundred and fifty of the most highly suppressed genes with a Detection score of Present or Marginal at the 1 week point (for the induced genes) or at the 0 h point (for the suppressed genes) were selected. Hierarchical clustering was performed using the Eisen Cluster and TreeView software package using the average linkage criterion [28].

### Validation by quantitative PCR (Q-PCR)

A 5 μg aliquot of total RNA was reverse transcribed using Superscript II reverse transcriptase (Invitrogen). A 10 ng aliquot of cDNA was used for each Q-PCR assay performed with the ABI Prism 7900 HT thermocycler (Applied Biosystems) using SYBR Green detection (Perkin-Elmer Applied Biosystems, Foster City, CA). Each assay was performed in triplicate using RNA samples extracted from 2-4 distinct populations of cells. The sequences of gene transcripts that correspond to the Affymetrix probe set were obtained from Genbank accession numbers listed on the Affymetrix website and primer sets corresponding to exon sequences for PCR amplifications were designed using the Primer Express software (Perkin-Elmer Applied Biosystems, Foster City, CA).

The expression of cyclophilin H was assessed in all samples, and did not alter significantly at different time-points, and therefore, was used to normalize starting cDNA concentrations, and the primers used for all Q-PCR are listed in Additional file 1: Table S1. Dissociation curves were performed as routine verification in order to check the primers for amplification of a single band, and template dilution standard curves (5-log range) were conducted with each primer set to verify a linear relationship between template concentration and Ct values (R^2 ^> 0.9). Absolute transcript concentrations were calculated using standard curves for each primer set. Relative ratios were then calculated for each cell type in a manner similar to the microarray ratios in order to allow relative comparison between data sets.

### Antibodies, immunoprecipitation and western blotting

All reagents have been described in earlier publications [10,16,20].

Ba/F3 BCR-ABL or TEL-PDGFRB cells were washed three times in 10 mM HEPES (pH 7.4)/Hanks balanced salts, and resuspended in RPMI-complete media or RPMI-complete media supplemented with IL-3 ± 1 μg/ml of Imatinib (Novartis). Lysates were prepared and analyzed by performing Western blot using the 4G10 anti-phosphotyrosine monoclonal antibody.

## Results

### BCR-ABL, TEL-PDGFRB and TEL-JAK2 trigger distinct gene expression changes at steady state

We examined changes in gene expression triggered by IL-3-stimulation and activation of BCR-ABL, TEL-PDGFRB or TEL-JAK2 at steady-state levels by using the MOE430A Affymetrix oligonucleotide array. In order to attribute changes in gene expression directly to BCR-ABL and TEL-PDGFRB kinase activity, we used Imatinib mesylate, which inhibits ABL, PDGFRB and the c-kit receptor (reviewed in [29] and [30]). Since Ba/F3 cells do not rely on ABL, PDGFRB or c-kit for their growth or survival, we reasoned that Imatinib would not interfere with the normal signaling pathways of Ba/F3 cells and that we could use this agent in order to regulate the activation of BCR-ABL and TEL-PDGFRB.

Ba/F3 cells were incubated in the absence of IL-3 for 5 h. Ba/F3 cells expressing BCR-ABL or TEL-PDGFRB were incubated without IL-3 and with Imatinib for 5 h. Previously, we observed that cells are arrested at the G0/G1 phase of the cell cycle with this treatment, and could be stimulated to enter the cell cycle by IL-3 or by kinase activation (Additional file 2: Figure S1 and Additional File 3: Figure S2).

After 5 h of incubation, Ba/F3, Ba/F3 BCR-ABL and Ba/F3 TEL-PDGFRB cells were activated. Total RNA was isolated from each cell line at 0, 8, 12, 24 h and 1 week. Total RNA was also isolated from Ba/F3 TEL-JAK2 cells that had been washed and incubated in IL-3-free media for 1 week to achieve steady-state factor-independence.

Unlike inhibiting BCR-ABL and TEL-PDGFRB with Imatinib, there is no inhibitor that can be used to specifically inhibit TEL-JAK2 without perturbing the normal growth and survival of Ba/F3 cells. Because Ba/F3 cells are IL-3-dependent and thus require Jak2, any conventional Jak2 inhibitor would disturb the normal growth and survival of Ba/F3 cells. To circumvent this issue, we compared the gene expression profile 1 week after Ba/F3 TEL-JAK2 cells were switched to factor-independence to the gene expression in Ba/F3 cells that had been depleted of cytokine for 5 h.

As we were mainly interested in the genes that are regulated at steady state, we identified genes that were induced or repressed by 2-fold or greater after 1 week. For the induced genes, we selected those genes that showed a Detection call of Present (*p*-value < 0.04) or Marginal (0.04 <*p*-value < 0.06) at the 1-week point. For the repressed genes, we selected those genes that showed a Detection call of Present or Marginal at the 0 h point.

**Figure 1:**
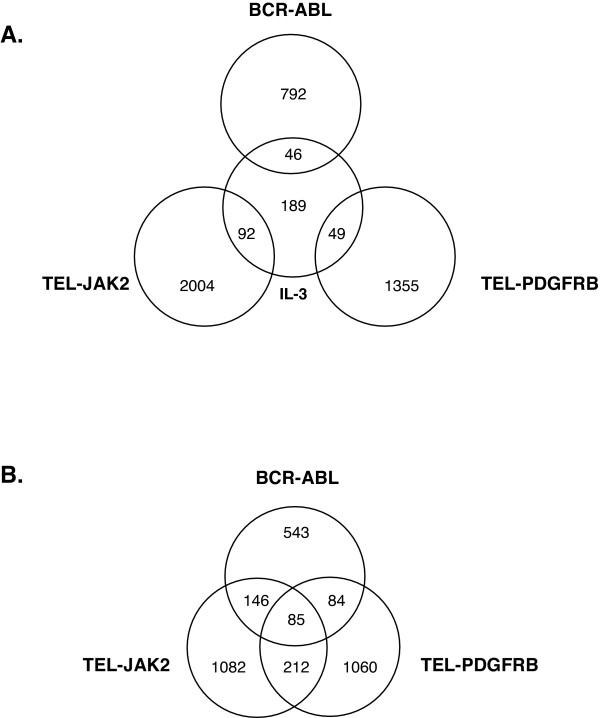
**Comparison of gene expression profiles among BCR-ABL, TEL-PDGFRB, TEL-JAK2 and IL-3**. Ba/F3 cells were washed and incubated in IL-3-depleted media for 5 h, then stimulated with IL-3 for 0 h or 1 week. Ba/F3 BCR-ABL cells and Ba/F3 TEL-PDGFRB cells washed and incubated in the media depleted of IL-3 and supplemented with Imatinib for 5 h, were washed and incubated in the absence of IL-3 and Imatinib for 0 h or 1 week. Ba/F3 TEL-JAK2 cells were washed and incubated in IL-3-depleted media for 1 week. Total RNA was collected at 0 h and 1 week time-points, and was used for microarray analysis. For all cell lines except for Ba/F3 TEL-JAK2 cells, changes in gene expression were calculated using the expression values at 1 week and the 0 h within each cell type. For Ba/F3 TEL-JAK2 cells, expression values obtained from Ba/F3 TEL-JAK2 cells at the 1 week time-point were compared to the expression values obtained from Ba/F3 cells at the 0 h time-point. The number of genes that were induced or suppressed by 2-fold or more with significant detection scores in each cell type as well as in overlapped regions is indicated in the Venn diagrams.

Using these criteria, all three fusion kinases induced distinct gene expression changes when compared to IL-3 at steady-state (Figure 1A). Similarly, when the fusion proteins were compared amongst themselves, the three fusion kinases induced distinct gene expression changes (Figure 1B). Downstream of each kinase fusion, we identified a subset of genes that are regulated by one fusion kinase but not by the other two kinases, as well as genes that are commonly regulated by all three fusions.

**Figure 2:**
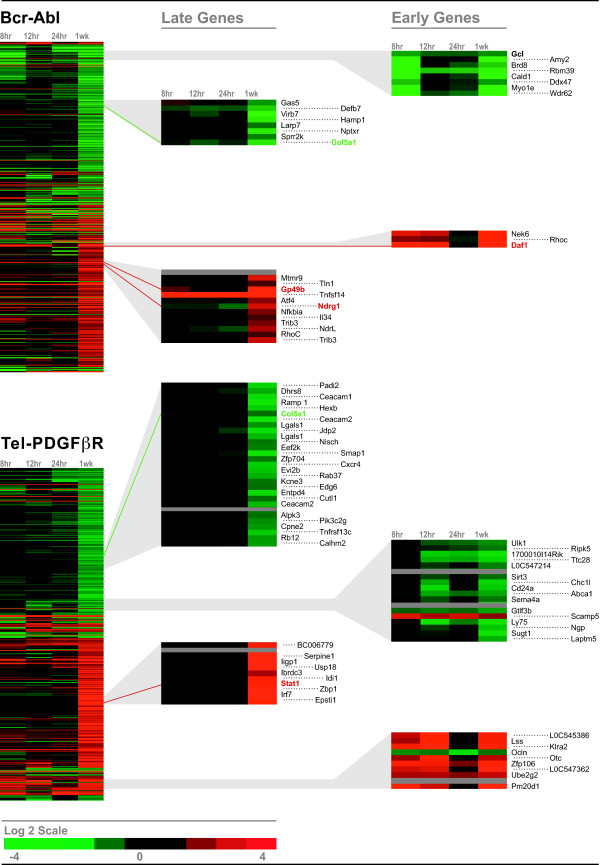
**Comparative gene expression profiling between genes regulated by BCR-ABL and genes regulated by TEL-PDGFRB**. Ba/F3 BCR-ABL cells and Ba/F3 TEL-PDGFRB cells were washed and incubated in media depleted of IL-3 and supplemented with Imatinib for 5 h. Cells were washed and incubated in the absence of IL-3 and Imatinib to activate the fusion kinases for 0, 8, 12, 24 h and 1 week. Total RNA was collected at each time-point and was used for microarray analysis. The 250 most highly induced and the 250 most highly suppressed genes at 1 week were identified in cells with BCR-ABL (*Top*) or TEL-PDGFRB (*Bottom*) fusion proteins. The 500 genes induced or suppressed by each fusion protein were hierarchically clustered using the Eisen Cluster and Tree View programs and are displayed on a log2 color scale, with red representing induced genes and green representing suppressed genes. *Grey *represents omitted data. Shown in the second and the third columns are the examples of genes that are regulated by 2-fold or greater within 24 h of activation ("Early Genes"), and after 24 h ("Late Genes"). Genes that were validated by Q-PCR are highlighted in red (induced) or green (suppressed) in the gene list.

Next, 500 genes were selected that were most highly induced or suppressed at steady state (250 induced and 250 suppressed genes) in cells expressing BCR-ABL or TEL-PDGFRB fusion protein. They were then clustered based on the changes in their gene expression at 8, 12, and 24 h time-points, using the Eisen hierarchical clustering algorithm (Figure 2). For each fusion kinase, genes were segregated into two main clusters that consisted of induced genes and suppressed genes. Within each cluster, we observed that a subset of genes displayed changes during the first 24 h of activation ("Early Genes"), while others displayed changes after 24 h of activation ("Late Genes"). Selected genes were validated by Q-PCR at one or more time-points (Figure 2, highlighted in red or green).

#### i) Genes regulated by BCR-ABL

Among the 543 genes induced or suppressed by BCR-ABL at steady state by two-fold or more, but not by TEL-PDGFRB or TEL-JAK2, four highly induced genes Cd55, Dok2, Mrvi1, and N-myc down-regulated gene-1 (Ndrg1) were selected for validation and were confirmed to be significantly induced (Table 1). Interestingly, Q-PCR validation/experiments suggests Dok2 and Mrvi1 were induced by TEL-PDGFRB, although both genes were initially identified to be uniquely induced by BCR-ABL in microarray experiments (see summary in Table 5). Moreover, Cd55 and Ndrg1 were confirmed to be suppressed by TEL-PDGFRB (see summary in Table 5).

**Table 1:** Genes regulated by BCR-ABL.

A. Microarray Analysis		
**Genes Regulated by BCR-ABL**		

	**Fold-Change**	**Gene Name**

Induced Genes	13.00	Complement component 3 (C3)
	
	12.12	Murine retrovirus integration site 1 (Mrvi1)*
	
	11.31	CD55 antigen (CD55)*
	
	4.29	N-myc downstream regulated gene 1 (Ndrg1)*
	
	3.48	Downstream of Tyrosine kinase 2 (Dok2)
	
	2.64	CCAAT enhancer binding protein, beta (Cebpb)
	
	2.46	A disintegrin and metalloprotease domain 8 (Adam8)
	
	2.46	Arrestin, beta 2 (Arrb2)
	
	2.40	Selectin, lymphocyte (Sell)
	
	2.30	Talin (Tln)
	
	2.30	Enolase 3, beta muscle (Eno3)

Suppressed Genes	3.03	Interleukin-15 (Il15)
	
	3.03	Neuronal pentraxin receptor (Nptxr)
	
	3.03	Ciliary neurotrophic factor (Cntf)
	
	2.64	Vimentin (Vmn)
	
	2.46	Suppression of tumorigenicity 7 (St7)
	
	2.30	Nuclear factor IX (Nfix)
	
	2.30	Growth arrest specific 5 (Gas5)
	
	2.14	Ribosomal protein S20 (Rps20)

**B. Validation by Quantitative PCR**		

**Gene Name**	**Mean Fold-Change**	***p*-Value**

Cd55	4.87 ± 1.31	0.02

Dok2	4.57 ± 1.82	0.07

Mrvi1	11.08 ± 0.76	0.0001

Ndrg1	4.92 ± 0.50	0.01

**(A) **Selected genes induced or suppressed by BCR-ABL by two-fold or more after 1 week of activation. Genes chosen for validation by Q-PCR are denoted by asterisks. **(B) **Q-PCR was performed in triplicate using RNA samples extracted from 2-4 distinct cell populations Mean fold-induction at 1 week compared to the expression at 0 h and standard error mean (SEM) are shown for each gene. A student *t*-test was performed to obtain P-values for the difference between the mean gene expression level at 0 h and 1 week. "I" and "S" denote "induced" and "suppressed", respectively

Although not selected for the validation study, Talin was previously reported to be induced in a BCR-ABL expressing cell line [31,32].

#### ii) Genes regulated by TEL-PDGFRB

Genes that are regulated by TEL-PDGFRB, but not by BCR-ABL or TEL-JAK2, are identified (Table 2). Interestingly, a large number of these genes have been previously reported to be IFN-stimulated genes (ISG) such as Cxcl10, Gbp1, Isg20, Stat1, Irf1 and Irf7 [33]. A subset of these genes (Vegf, Pim1 and Isg20) was also induced upon IL-3 stimulation and all three tyrosine kinases, suggesting that these targets may be bona fide cytokine-regulated transcripts. However, TEL-PDGFRB stimulated the expression of 25 ISGs (summarized in Table 3). Among the TEL-PDGFRB-regulated genes, Cxcl10, Gbp1 and Stat1 were selected for validation by Q-PCR.

**Table 2:** Genes regulated by TEL-PDGFRB.

A. Microarray Analysis		
**Genes Regulated by TEL-PDGFRB**		

	**Fold-Change**	**Gene Name**

Induced Genes	234.50	Chemokine (C-X-C motif) ligand 10 (Cxcl10)
	
	103.12	Guanylate nucleotide binding protein 1 (Gbp1)
	
	64.00	Immune responsive gene 1 (Irg1)
	
	42.22	Ubiquitin specific protease 18 (Usp18)
	
	19.00	Signal transducer & activator of transcription 1 (Stat1)
	
	14.55	Interferon regulatory factor 7 (Irf7)
	
	7.46	Leukemia inhibitory factor (Lif)
	
	6.96	Jun oncogene (Jun)
	
	5.84	Vascular endothelial growth factor A (Vegfa)

Suppressed Genes	2.83	Gelsolin (Gsn)
	
	2.64	Elk3, member of Ets oncogene family (Elk3)
	
	2.64	Cyclin I (Ccni)
	
	2.64	Cyclin-dependent kinase inhibitor 2c (Cdkn2c)
	
	2.46	Leukotriene A4 hydrolase (Lta4h)
	
	2.46	Adducin 1, alpha (Add1)
	
	2.46	Neutrophilic granule protein (Ngp)
	
	2.46	Eukaryotic elongation factor-2 kinase (Eef2k)
	
	2.46	EGF-like module containing, mucin-like, hormone receptor-like sequence 1 (Emr1)

**B. Validation by Quantitative PCR**		

**Gene Name**	**Mean Fold-Change**	***p*-Value**

Cxcl10	61.55 ± 20.98	0.02

Stat1	2.70 ± 0.45	0.009

Gbp1	83.47 ± 20.89	0.008

**(A) **Selected genes that were induced or suppressed by TEL-PDGFRB by two-fold or more after 1 week of activation. Genes chosen for validation by Q-PCR are denoted by asterisk. **(B) **Q-PCR was performed in triplicate using RNA samples extracted from 4-5 distinct cell populations. Mean fold-induction at 1 week compared to the expression at 0 h, and SEM are shown for each gene. A student *t*-test was performed to obtain p-values for the difference between the mean gene expression level at 0 h and 1 week

**Table 3:** Induction of Interferon-Stimulated Genes downstream of TEL-PDGFRB.

Gene Description	Fold-induction
Chemokine (C-X-C motif) ligand 10 (Cxcl10)	234.5

Guanylate nucleotide binding protein 1 (Gbp1)	103.1

Guanylate nucleotide binding protein 2 (Gbp2)	53.0

Signal transducer and activator of transcription 1 (Stat1)	19.0

Interferon-regulatory factor 7 (Irf7)	14.6

Myxovirus-resistance 1 (Mx1)	10.1

Isg15 ubiquitin-like modifier (Isg15)	8.2

HLA, Class I, G (Hla-g)	7.2

Proteasome subunit, beta type 9 (Psmb9)	6.3

Proteasome subunit, beta type 10 (Psmb10)	5.7

Lectin, galactoside-binding, soluble, 3 binding protein (Lgals3bp)	4.6

Caspase 4 (Casp4)	4.4

Interferon-induced protein with tetratricopeptide repeats 2 (Ifit2)	4.3

Nicotinamide phosphoribosyltransferase (Nampt)	4.3

Interferon-regulatory factor 1 (Irf1)	4.2

Tyrosyl tRNA synthetase (Yars)	4.1

Tripartite motif containing 21	3.7

N-myc and Stat interactor (Nmi)	3.3

Pml transcription factor (Pml)	3.1

Tryptophanyl tRNA synthetase (Wars)	2.8

Eif2 alpha kinase 3 (Eif2ak3)	2.6

Proteasome subunit, beta type 8 (Psmb8)	2.4

Proteasome 28 subunit, alpha (Psme1)	2.3

Interferon regulatory factor 9 (Irf9)	2.2

Protein phosphatase 5, catalytic subunit (Ppp5c)	2.2

Genes regulated by TEL-PDGFRB that were previously reported to be ISGs were identified. Data represent the fold-changes in gene expression at steady state obtained from the microarray experiment

All three genes were induced by TEL-PDGFRB as confirmed by Q-PCR (Table 2). Interestingly, the expression of Cxcl10 was similarly elevated downstream of BCR-ABL, whereas the induction of Stat1 and Gbp1 was observed uniquely downstream of TEL-PDGFRB (see summary in Table five).

#### iii) Genes regulated by TEL-JAK2

Genes regulated by TEL-JAK2 at 1 week, but not by BCR-ABL or TEL-PDGFRB, were identified and the abridged list is shown in Table 4. Of these, Cited2 and Bnip3 were found in our previous cDNA microarray analysis to be suppressed during the first 12 h after Ba/F3 TEL-JAK2 cells are switched to factor-independence (data not shown). Findings from the present study demonstrate that these transcripts may remain suppressed at steady state.

**Table 4:** Genes regulated by TEL-JAK2 at steady state.

Microarray Analysis		
**Genes Regulated by TEL-JAK2**		

	**Fold-Change**	**Gene Name**

Induced Genes	3.90	0-6-methylguanine-DNA methyltransferase (Mgmt)
	
	3.20	Serum deprivation response (Sdpr)
	
	3.18	Growth arrest specific 5 (Gas5)
	
	3.13	Immediate early response 2 (Ier2)
	
	2.74	Cbp/p300 interacting transactivator 2 (Cited2)
	
	2.69	E2f transcription factor 6 (E2f6)
	
	2.38	Pannexin 1 (Panx1)
	
	2.17	Myosin VIIa (Myo7a)
	
	2.12	Tnf receptor-associate factor 5 (Traf5)
	
	2.12	Cyclin-dependent kinase inhibitor 2a (Cdkn2a)
	
	2.00	Rab33b, member of Ras oncogene family (Rab33b)

Suppressed Genes	6.81	Chemokine (C-C) receptor 1 (Ccr1)
	
	3.30	Apoptosis inhibitory protein 5 (Api5)
	
	2.98	Bcl2 adenovirus E1b 19 kDa interacting protein 3-like (Bnip3)
	
	2.85	Runt-related transcription factor 1 (Runx1)
	
	2.68	Lim only 4 (Lmo4)
	
	2.61	Chemokine (CXC motif) ligand 13 (Cxcl13)
	
	2.47	Axin1 (Axin1)
	
	2.17	Rho family GTPase RhoA (RhoA)
	
	2.12	Chemokine (C-C motif) receptor 1 (Ccr1)

Ba/F3 cells were washed and incubated in IL-3-depleted media for 5 h and total RNA was collected as reference RNA. Ba/F3 TEL-JAK2 cells were washed three times and incubated in IL-3-depleted media for 1 week and total RNA was collected. Microarray analysis was performed and changes in the gene expression between the reference RNA and the RNA collected from Ba/F3 TEL-JAK2 cells were calculated. Genes that are induced or suppressed by two fold or greater were identified.

#### iv) Genes commonly regulated by BCR-ABL, TEL-PDGFRB and TEL-JAK2

**Figure 3:**
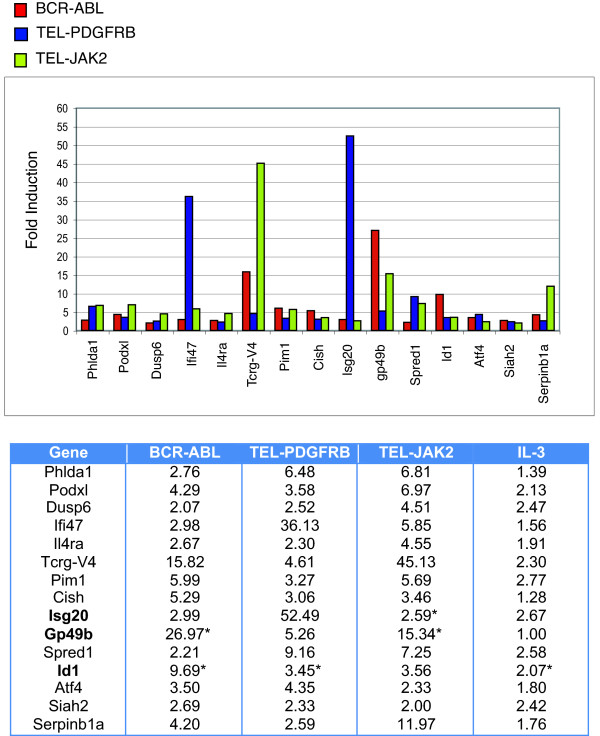
**A subset of genes is commonly induced by BCR-ABL, TEL-PDGFRB and TEL-JAK2**. Ba/F3 BCR-ABL cells and Ba/F3 TEL-PDGFRB cells were washed and incubated in the media depleted of IL-3 and supplemented with Imatinib for 5 h. Cells were washed and incubated in the absence of IL-3 and Imatinib to activate the fusion kinases for 0 h and 1 week. Ba/F3 TEL-JAK2 cells were washed and incubated in IL-3-depleted media for 1 week, and Ba/F3 cells were washed and incubated in IL-3-depleted media for 5 h to be used as a reference for Ba/F3 TEL-JAK2 cells. Total RNA was collected from each cell line and microarray analysis was performed using a mouse oligonucleotide array. For cells expressing BCR-ABL or TEL-PDGFRB, changes in gene expression were calculated using the expression values at 1 week and 0 h. For Ba/F3 TEL-JAK2 cells, expression values obtained from Ba/F3 TEL-JAK2 cells at the 1-week time point were compared to the expression values obtained from Ba/F3 cells after 5 h of IL3-depletion. Fold-inductions of gene expression are indicated in the table (*Bottom*) and are displayed in a bar-graph (*Top*). Asterisks denotes genes that were validated by Q-PCR. **Phlda1**, Pleckstrin homology-like domain, family A, member 1; **Pdxl**, podocalyxin; **Dusp6**, dual specificity phosphatase 6; **Ifi47**, interferon gamma inducible protein, 47 kDa; **Il4ra**, interleukin 4 receptor, alpha; **Tcrg-V4**, T-cell receptor gamma, variable 4; **Pim1**, proviral integration site 1; **Cish**, cytokine inducible SH2-containing protein; **Isg20**, interferon stimulated protein 20; **gp49b**, C3H gp49b; **Spred1**, Sprouty protein with EVH-1 domain 1, related sequence; **Id1**, transcription factor Id1; **Atf4**, activating transcription factor 4; **Siah2**, Seven in absentia 2; **Serpinb1a**, serine(or cysteine) proteinase inhibitor, clade B (ovalbumin) 1a.

**Figure 4:**
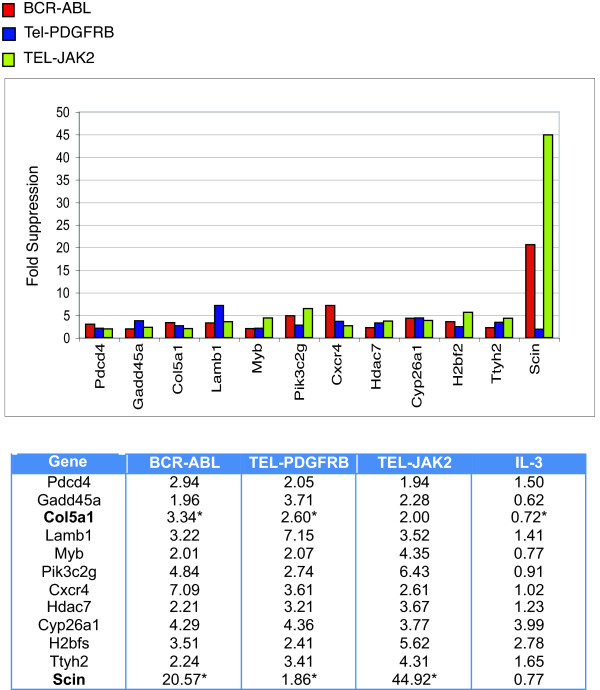
**A subset of genes is commonly suppressed by BCR-ABL, TEL-PDGFRB and TEL-JAK2**. Genes induced or suppressed by BCR-ABL, TEL-PDGFRB and TEL-JAK2 by 2-fold or greater were identified. Fold-suppressions of gene expression are indicated in the table (*Bottom*) and are displayed in a bar-graph (*Top*). Asterisks denote genes that were validated by Q-PCR. **Pdcd4**, programmed cell death 4; **Gadd45a**, growth arrest and DNA-damage-inducible 45a; **Col5a1**, procollagen, type V, alpha1; **Lamb1**, laminin B1 subunit; **Myb**, myeloblastosis oncogene; **Pik3c2g**, PI3-kinase, C2 domain containing gamma polypeptide; **Hdac7**, histone deacetylase 7; **Cyp26a1**, Cytochrome P450, family 26, subfamily a, polypeptide 1; **H2bfs**, H2B histone family, member 2; **Ttyh2**, tweety homolog 2; **Scin**, scinderin.

Finally, we identified a subset of genes that were regulated by all three fusion kinases. Id1, gp49b, Col5a1, Scinderin and Isg20 were selected for validation by Q-PCR (Figures 3 &4). Among the five transcripts, only Scinderin was commonly suppressed by all three fusions in the Q-PCR analysis, while the other four transcripts displayed overlapping regulation between two of the three fusion kinases (Table 5).

**Table 5:** Summary of genes regulated by IL-3, BCR-ABL, TEL-PDGFRB and TEL-JAK2 at steady-state (1 week).

Gene Name	IL-3	BCR-ABL	TEL-PDGFRB	TEL-JAK2
**Dok2**	-	Up*	-	-

**Daf1**	-	Up	Down	-

**Ndrg1**	-	Up	Down	-

**Stat1**	-	-	Up	-

**Gbp1**	-	-	Up	?

**Isg20**	-	-	Up	Up

**Id1**	Up*	Up	Up*	-

**Mrvi1**	-	Up	Up	-

**Gp49b**	Up	Up*	-	Up

**Cxcl10**	-	Up	Up	Up

**Scin**	-	Down	Down	Down

**Col5a1**	Down	Down	Down*	-

All genes were validated by Q-PCR using RNA samples extracted from 2-5 distinct cell populations. Data represent mean fold-change in gene expression at 1 week compared to the expression at 0 h and SEM. All changes in gene expression were associated with *p*-value < 0.05 as determined by a student *t*-test unless indicated by asterisk, which denotes *p*-value < 0.1. Dotted line (-) indicates no significant change

In addition, Cxcl10 was confirmed to be induced by all three fusions even though it was initially identified as a TEL-PDGFRB-regulated gene (Tables 2 & 5).

#### v) BCR-ABL triggers early gene expression changes of Scinderin and Id1

We examined the kinetics of the regulation of a subset of transcripts (Id1, Scinderin, Stat1, Col5a1, Cxcl10 and gp49b) that had been confirmed by Q-PCR to be significantly regulated by BCR-ABL or TEL-PDGFRB fusion protein after 1 week. As we have no means to inhibit TEL-JAK2-mediated proliferation, the analysis of the kinetics was performed only in Ba/F3 cells and Ba/F3 cells expressing BCR-ABL or TEL-PDGFRB.

**Figure 5:**
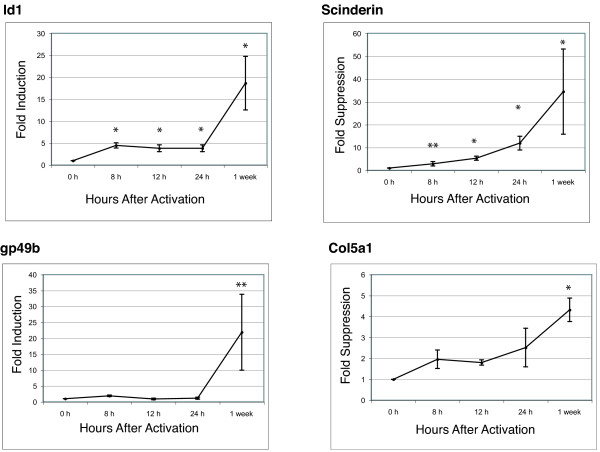
**Regulation of Id1, Scinderin, Gp49b and Col5a1 by BCR-ABL**. Total RNA was extracted at 0, 8, 12, and 24 h after BCR-ABL activation in Ba/F3 BCR-ABL cells. Q-PCR was performed and relative gene expression was calculated with respect to the expression level at 0 h. Line graphs represent mean fold-changes and SEM in the gene expression obtained from four separate experiments. The student *t*-test was performed to identify statistically significant changes at 95% (*) or 90% (**) confidence level.

After 0, 8, 12, and 24 h of kinase activation, total RNA was collected and Q-PCR was performed. Despite the large variance between replicate experiments, we consistently observed that the BCR-ABL-mediated induction of Id1 transcript occurs within the first 8 h after BCR-ABL activation in Ba/F3 BCR-ABL cells (Figure 5). We also assessed the changes in the Id1 transcript level during the first 24 h after TEL-PDGFRB activation. Although a trend of gene induction was observed during the first 24 h, this trend was highly variable, and we were unable to detect a consistent induction at early time-points downstream of TEL-PDGFRB (data not shown).

In contrast to Id1, the gp49b transcript did not display any significant change downstream of BCR-ABL during the first 24 h (Figure 5 & data not shown). Similarly, the expression of Cxcl10 did not change substantially downstream of BCR-ABL or TEL-PDGFRB even though it is induced by all three fusions kinases at steady state (Table 5 & data not shown).

Among the suppressed genes, Scinderin was significantly suppressed by all three fusions and the suppression occurred within the 24 h of activation of BCR-ABL or TEL-PDGFRB (Figure 5 & data not shown). Finally, Col5a1 did not exhibit any significant change at the earlier time-points downstream of BCR-ABL or TEL-PDGFRB (Figure 5 & data not shown).

Temporal regulation of Stat1 was also assessed downstream of TEL-PDGFRB, and it was not significantly altered at the earlier time-points (data not shown).

## Discussion and conclusions

BCR-ABL, TEL-PDGFRB and TEL-JAK2 are recurrent chromosomal translocations associated with distinct forms of leukemia that differ in the target cell and in disease aggressiveness. All three fusion proteins activate similar signaling pathways involving MAP kinases, Stat proteins and PI3K in hematopoietic cell lines. Our current understanding of the cytosolic signaling pathways of these fusion proteins alone cannot explain the basis for these differences, and we reasoned that examination of gene expression changes regulated by the three fusions might provide insights in this regard.

The results from the oligonucleotide array analysis demonstrate that the three tyrosine kinase fusions trigger both overlapping and distinct gene expression changes, and these results were confirmed by Q-PCR (Table 5). In addition, we demonstrated that TEL-PDGFRB induced a large number of ISG.

Because we can attribute gene expression changes downstream of BCR-ABL and TEL-PDGFRB directly to these kinases, we chose 12 genes regulated by BCR-ABL or TEL-PDGFRB for Q-PCR validation based on the fold-change in their expression, the Detection score in the gene expression as predicted by MAS 5.0, and their cellular function. As we were interested in the genes that could potentially contribute to leukemogenesis, we selected those that modulate cell proliferation and differentiation.

Validation by Q-PCR revealed that Dok2, Isg20, Id1, Mrvi1, gp49b, Cxcl10, Scinderin and Col5a1 display an overlapping regulation among the three fusion kinases by 2-fold or greater at steady-state (Table 5). In particular, Cxcl10 and Scinderin are commonly regulated by all three fusions. Cd55 and Ndrg1 are suppressed by TEL-PDGFRB, whereas they are induced by BCR-ABL. Finally, Stat1 and Gbp1 are uniquely induced by TEL-PDGFRB.

Most studies that examine the gene expression changes associated with BCR-ABL have compared the normal hematopoietic progenitors to the CML progenitors [32,34-38]. A caveat of this approach is that it is difficult to attribute genetic changes directly to BCR-ABL as they may have arisen from secondary mutations. The use of Imatinib allowed us to link changes in gene expression directly to BCR-ABL and TEL-PDGFRB, and to examine the kinetics of gene regulation. Kinetic analysis revealed that a distinct subset of genes was regulated within the first 8 h of kinase activation (e.g. Id1, Scinderin), while others were regulated after 24 h (e.g. Stat1, Cxcl10, gp49b and Col5a1). The late changes in the expression of genes such as Stat1, Cxcl10, gp49b and Col5a1 may be attributable to the relatively small extent of changes in gene expression at the earlier time-points, and it is possible that early changes occurred but were not detectable in our system.

Many of the genes identified in our study are associated with regulation of cell migration, proliferation and differentiation, all of which are known to be perturbed during leukemogenesis. Considering the functions of the validated genes, the finding that these genes are regulated downstream of the three fusions suggest that they may play important roles during leukemia development.

Cxcl10, gp49b and Mrvi1 are membrane proteins up-regulated in our experiments. Cxcl10, is a member of the chemokine family, that plays an important role in regulating movement and retention of hematopoietic progenitor cells within the bone marrow microenvironment. Chemokines have been implicated in Imatinib resistance [39] and as a potential mechanism for effective interferon treatment [40]. CML cells fail to respond to at least two different chemokines: macrophage inflammatory protein (MIP)-1α and stromal-derived factor (SDF)-1 [41-44]. Moreover, it was demonstrated that BCR-ABL both positively and negatively regulates SDF-1-mediated signaling suggesting that BCR-ABL autonomously regulates the cell migration and retention of hematopoietic progenitors in the marrow [43,45].

gp49b, also known as leukocyte immunoglobulin-like receptor, subfamily B, member 4 (Lilrb4), is a ITIM-containing, Ig-like receptor expressed on mast cells that plays an inhibitory role in IgE- or cytokine-dependent mast cell activation and inflammation [46-48]. gp49b was found to be up-regulated at the mRNA level by G-CSF stimulation in multiple myeloid cell lines [49] and its related member, LILRB3 has been reported to be highly induced in CML-chronic phase samples in microarray experiments [32]. While we observed a transient induction of gp49b around the 8 h time-point in some instances (Figure 5), this transient induction was not sustained at 24 h. Given that gp49b is significantly elevated at 1 week, our findings suggest that the induction of gp49b may be subsequent to changes that occur early in response to BCR-ABL activation.

Col5a1 is a member of Type V collagen family, which mediates cell adhesion by binding preferentially to α_2_β_1 _integrin [50]. Although no direct evidence exists between Type V collagens and leukemia, adhesion receptors play a critical role during hematopoiesis as they relay the external cues to the cells, and CML cells are defective in cell adhesion to the bone marrow stroma and extracellular matrix. Moreover, Col1a1 and Col4a4, have been previously reported to be highly suppressed in CML chronic phase samples in cDNA microarray experiments [32,51-56]. Col5a1 is a HIF-1α target gene, and the expression of HIF-1α has been shown to be a positive prognostic factor in lymphoma [57]. In our study, Col5a1 was significantly suppressed at steady state by both BCR-ABL and TEL-PDGFRB, suggesting that its suppression may contribute to leukemia caused by these two fusions.

Several signal transducers were regulated by activated tyrosine kinases in our experiments including Dok2 and Scinderin. Dok2 was consistently elevated downstream of BCR-ABL and TEL-PDGFRB. Dok1 and Dok2 exhibit increased tyrosine phosphorylation in p210^BCR-ABL^-expressing cell lines [58] and tyrosine phosphorylation of Dok2 was significantly down regulated in Mo7e/p210^BCR-ABL ^cells treated with Imatinib [59]. Dok2 mediates cytoskeletal activity by its direct interaction with c-Abl, resulting in increased c-Abl tyrosine phosphorylation and kinase activity [60]. In contrast to our study, Nowicki et al. observed a strong down-regulation of the Dok2 transcript in the bone marrow cells of patients with CML at blast crisis [32]. The study by Nowicki et al., however, has a few caveats, which may explain the discrepancy between our results. Firstly, the finding was made by cDNA microarray analysis, and the result was not validated by a quantitative assay. Secondly, the authors compared a highly heterogeneous population of CML samples from peripheral blood mononuclear cells to fully differentiated normal control cells. On the other hand, the discrepancy between these reports may reflect differences in the experimental systems employed in each of these studies. In addition, two studies demonstrated that a compound Dok1/Dok2 knockout mouse developed a CML-like disease [61,62]. We observed no difference in Dok1 expression in Ba/F3 BCR-ABL cells in our microarray experiments. Additionally, the role of Dok2 downstream of TEL-PDGFRB remains to be explored.

Scinderin (adseverin) was highly suppressed by all three fusions at steady state, and this occurred as early as 8 h after BCR-ABL or TEL-PDGFRB activation. Scinderin is a Ca^2+^-dependent actin severing protein, expressed in normal megakaryocytes and platelets that controls actin filament length [63]. The role of Scinderin is crucial in regulating cortical F-actin to mediate secretion including the release of platelets from mature megakaryocytes. Proteomic analysis of Ba/F3 BCR-ABL and Ba/F3 TEL-PDGFRB cells revealed that Scinderin is down-regulated by BCR-ABL, but not by TEL-PDGFRB, however, the result was not validated by Western blotting [64]. Expression of Scinderin in MEG-01 cells promoted platelet formation and inhibited tumor formation when MEG-01/Scinderin cells were injected into nude mice. MEG-01 is a CML cell line, however, the role of scinderin in BCR-ABL function was not examined in this study [65]. Nevertheless, our results lead to the hypothesis that suppression of scinderin may represent a mechanism by which BCR-ABL, TEL-PDGFRB and TEL-JAK2 promote leukemic transformation.

ID1, (Inhibitor of DNA binding 1), has been previously shown to be induced in samples from CML patients in chronic phase of the disease suggesting its potential role in leukemia development, and our data further support this hypothesis [32]. Another study confirmed that Id1 was up-regulated in response to various oncogenic tyrosine kinases, including BCR-ABL, TEL-ABL, TEL-PDGFRB, TEL-JAK2, TEL-TRKC, and FLT3-ITD [66]. Moreover, inhibition of each of those kinases in leukemic cell lines resulted in significant reductions in Id1 expression, suggesting that Id1 is a common downstream target of deregulated kinases. Id proteins are dominant negative regulators of Helix-loop-helix transcription factors that govern growth and development in mammals [67]. Previously, it has been shown that Id1 can be regulated by C/EBPβ [68], and we detected an elevated level of the Cebpb transcript 1 week after BCR-ABL activation. Other immediate early gene(s) may be involved in mediating the observed induction of Id1.

Finally, Stat1 and Gbp1 were uniquely induced by TEL-PDGFRB. Tyrosine phosphorylation of Stat1 and DNA binding has been previously observed downstream of BCR-ABL [5,7], TEL-PDGFRB [11] and TEL-JAK2 [10]. Interestingly, the induction of Stat1 occurred relatively late (after 24 h) in response to TEL-PDGFRB activation. Given that TEL-PDGFRB also induced a large number of IFN-regulated genes including Gbp1, it is tempting to speculate that there may exist an autocrine loop of IFN-γ, and that this in turn induces the transcription of Stat1 and Gbp1. In support of this hypothesis, Ohmine et al. reported the elevated levels of transcription of IFN-related genes including IFN-γ and IFN-γ receptor two in CML cells from patients in the chronic phase [34]. Moreover, Advani et al. observed that transcription of a group of IFN-inducible genes was significantly increased in primary mouse bone marrow cells expressing p185 BCR-ABL compared to those expressing p210 BCR-ABL, and this was accompanied by an induction of IFN-γ [36]. However, neither IFN-γ nor its receptors were induced at any of the time points examined in our experiments. It will be interesting to determine whether Stat1 exerts a direct transcriptional effect in TEL-PDGFRB transformed cells, independent of IFN-γ. In terms of its role during leukemogenesis, however, Stat1 was shown to be dispensable in a myeloproliferative disease induced by Tel-PDGFRB [69].

In summary, our study revealed that BCR-ABL, TEL-PDGFRB and TEL-JAK2 trigger distinct, yet overlapping changes in gene expression. Several genes that are known to be involved in regulating cell migration, proliferation and differentiation were significantly induced or suppressed at steady state as determined by Q-PCR, giving rise to a hypothesis that their regulation downstream of the three fusions may contribute to leukemogenesis. Further characterization of the genes identified in our study may enhance our understanding of the molecular basis underlying the specificity of diseases caused by the three fusion proteins.

## Competing interests

The authors declare that they have no competing interests.

## Authors' contributions

HK, SY and KH performed experiments, SD and DLB directed the study, HK, LCG, JDJ and DLB wrote the paper. All authors read and approved the final manuscript.

## Pre-publication history

The pre-publication history for this paper can be accessed here:

http://www.biomedcentral.com/1471-2407/11/528/prepub

## Supplementary Material

Additional file 1 **Table S1**. Primer sequences used for Q-PCR. Click here for file

Additional file 2 **Figure S1**. Imatinib inhibits kinase activities of BCR-ABL and TEL-PDGFRB. Ba/F3 cells expressing BCR-ABL (A) or TEL-PDGFRB (B) were washed and incubated in the presence of IL-3 and Imatinib (blue), or in the absence of IL-3 and in the presence of Imatinib (red) for 48 h. The X-axes represent a range of Imatinib concentrations tested. The green lines represent a vehicle control where cells were incubated in the absence of IL-3 and increasing amounts of DMSO, which was used to dissolve Imatinib. The arrows indicate the doses selected for culturing Ba/F3 cells expressing BCR-ABL or TEL-PDGFRB C, D; Lysates were collected in an IL-3-dependent state (IL3+/Imat+) and factor-independent state (IL3-/Imat-) and Western blotting was performed using an anti-phosphotyrosine monoclonal antibody to detect phosphorylated forms of BCR-ABL (C). Phosphorylated TEL-PDGFRB was detected by immunoprecipitation with a Tel-antibody, followed by Western blotting using 4 G10 (D). For IL-3-dependent state, Ba/F3 TEL-PDGFRB cells and Ba/F3 BCR-ABL cells were incubated in the presence of 0.1 or 1 mg/ml of Imatinib, respectively. Click here for file

Additional file 3 **Figure S2**. Cell cycle changes induced by BCR-ABL, TEL-PDGFRB and IL-3. Ba/F3, Ba/F3 BCR-ABL and Ba/F3 TEL-PDGFRB cells growing in RPMI and IL-3 were washed. Ba/F3 cells were resuspended in RPMI complete media and Ba/F3 BCR-ABL and Ba/F3 TEL-PDGFRB cells were resuspended in RPMI complete + Imatinib for 5 h to induce cell cycle arrest. Ba/F3 cells were stimulated with IL-3 for the indicated lengths of time. Cells expressing BCR-ABL or TEL-PDGFRB were washed to remove Imatinib, and incubated in RPMI media depleted of IL-3 and Imatinib in order to activate BCR-ABL and TEL-PDGFRB for the indicated lengths of time. Cells were harvested at each time-point and analyzed for changes in the G0/G1 fraction and the S fraction. Results are representative of data obtained from three independent experiments. Click here for file

## References

[B1] Konopka J Watanabe S Witte O An alteration of the human c-abl protein in K562 leukemia cells unmasks associated tyrosine kinase activity Cell 37 1035 1042 10.1016/0092-8674(84)90438-0 6204766

[B2] Golub TR Barker GF Lovett M Gilliland DG Fusion of PDGF receptor b to a novel ets-like gene, tel, in chronic myelomonocytic leukemia with t(5;12) chromosomal translocation Cell 1994 77 307 316 10.1016/0092-8674(94)90322-0 8168137

[B3] Lacronique V Boureux A Valle VD Poirel H Quang CT Mauchauffe M Berthou C Lessard M Berger R Ghysdael J A TEL-JAK2 fusion protein with constitutive kinase activity in human leukemia Sci 1997 278 5341 1309 1312 10.1126/science.278.5341.1309 9360930

[B4] Peeters P Raynaud SD Cools J Wlodarska I Grosgeorge J Philip P Monpoux F Van Rompaey L Baens M Van den Berghe H Fusion of TEL, the ETS-variant gene 6 (ETV6), to the receptor-associated kinase JAK2 as a result of t(9;12) in a lymphoid and t(9;15;12) in a myeloid leukemia Blood 1997 90 7 2535 2540 9326218

[B5] Carlesso N Frank DA Griffin JD Tyrosyl phosphorylation and DNA binding activity of signal transducers and activators of transcription (STAT) proteins in hematopoietic cell lines transformed by Bcr/Abl J Exp Med 1996 183 3 811 820 10.1084/jem.183.3.811 8642285 PMC2192351

[B6] Frank DA Varticovski L BCR/abl leads to the constitutive activation of Stat proteins, and shares an epitope with tyrosine phosphorylated Stats Leukemia 1996 10 11 1724 1730 8892675

[B7] Ilaria RL Jr Van Etten RA P210 and P190(BCR/ABL) induce the tyrosine phosphorylation and DNA binding activity of multiple specific STAT family members J Biol Chem 1996 271 49 31704 31710 10.1074/jbc.271.49.31704 8940193

[B8] Chai S Nichols G Rothman P Constitutive activation of JAKs and STATs in BCR-Abl-expressing cell lines and peripheral blood cells derived from leukemic patients J Immunol 1997 159 10 4720 4728 9366395

[B9] Shuai K Halpern J Hoeve Jt Rao X Sawyers CL Constitutive activation of STAT5 by the BCR-ABL oncogene in chronic myelogenous leukemia Oncogene 1996 13 247 254 8710363

[B10] Ho JM Beattie BK Squire JA Frank DA Barber DL Fusion of the ets transcription factor TEL to Jak2 results in constitutive Jak-Stat signaling Blood 1999 93 12 4354 4364 10361134

[B11] Wilbanks A Mahajan S Frank D Druker B Gilliland D Carroll M TEL/PDGFbetaR fusion protein activates STAT1 and STAT5: a common mechanism for transformation by tyrosine kinase fusion proteins Exp Hematol 2000 28 584 593 10.1016/S0301-472X(00)00138-7 10812249

[B12] Sternberg DW Tomasson MH Carroll M Curley DP Barker G Caprio M Wilbanks A Kazlauskas A Gilliland DG The TEL/PDGFbeta R fusion in chronic myelomonocytic leukemia signals through STAT5-dependent and STAT5-independent pathways Blood 2001 98 12 3390 3397 10.1182/blood.V98.12.3390 11719379

[B13] Nelson EA Walker SR Weisberg E Bar-Natan M Barrett R Gashin LB Terrell S Klitgaard JL Santo L Addorio MR The STAT5 inhibitor pimozide decreases survival of chronic myelogenous leukemia cells resistant to kinase inhibitors Blood 2011 117 12 3421 3429 10.1182/blood-2009-11-255232 21233313 PMC3069678

[B14] Mahlmann S McLaughlin J Afar DE Mohr R Kay RJ Witte ON Dissection of signaling pathways and cloning of new signal transducers in tyrosine kinase-induced pathways by genetic selection Leukemia 1998 12 12 1858 1865 10.1038/sj.leu.2401231 9844916

[B15] Sattler M Mohi MG Pride YB Quinnan LR Malouf NA Podar K Gesbert F Iwasaki H Li S Van Etten RA Critical role for Gab2 in transformation by BCR/ABL Cancer Cell 2002 1 5 479 492 10.1016/S1535-6108(02)00074-0 12124177

[B16] Ho JM Nguyen MH Dierov JK Badger KM Beattie BK Tartaro P Haq R Zanke BW Carroll MP Barber DL TEL-JAK2 constitutively activates the extracellular signal-regulated kinase (ERK), stress-activated protein/Jun kinase (SAPK/JNK), and p38 signaling pathways Blood 2002 100 4 1438 1448 12149229

[B17] Atfi A Prunier C Mazars A Defachelles AS Cayre Y Gespach C Bourgeade MF The oncogenic TEL/PDGFR beta fusion protein induces cell death through JNK/SAPK pathway Oncogene 1999 18 26 3878 3885 10.1038/sj.onc.1202734 10445851

[B18] Wheadon H Welham MJ The coupling of TEL/PDGF{beta}R to distinct functional responses is modulated by the presence of cytokine: involvement of mitogen-activated protein kinases Blood 2003 102 4 1480 1489 10.1182/blood-2002-09-2974 12714513

[B19] Skorski T Bellacosa A Nieborowska-Skorska M Majewski M Martinez R Choi JK Trotta R Wlodarski P Perrotti D Chan TO Transformation of hematopoietic cells by BCR/ABL requires activation of a PI-3 k/Akt-dependent pathway Embo J 1997 16 20 6151 6161 10.1093/emboj/16.20.6151 9321394 PMC1326299

[B20] Nguyen MH Ho JM Beattie BK Barber DL TEL-JAK2 mediates constitutive activation of the phosphatidylinositol 3'-kinase/protein kinase B signaling pathway J Biol Chem 2001 276 35 32704 32713 10.1074/jbc.M103100200 11435425

[B21] Dierov J Xu Q Dierova R Carroll M TEL/platelet-derived growth factor receptor beta activates phosphatidylinositol 3 (PI3) kinase and requires PI3 kinase to regulate the cell cycle Blood 2002 99 5 1758 1765 10.1182/blood.V99.5.1758 11861293

[B22] Naughton R Quiney C Turner SD Cotter TG Bcr-Abl-mediated redox regulation of the PI3K/AKT pathway Leukemia 2009 23 8 1432 1440 10.1038/leu.2009.49 19295548

[B23] Ren R Mechanism of Bcr-Abl in the pathogenesis of chronic myelogenous leukaemia Nat Rev Cancer 2005 5 172 183 10.1038/nrc1567 15719031

[B24] Chalandon Y Schwaller J Targeting mutated protein tyrosine kinases and their signaling pathways in hematologic malignancies Haematologica 2005 90 949 968 15996933

[B25] Chalandon Y Schwaller J Targeting mutated protein tyrosine kinases and their signaling pathwyas in hematologic malignancies Haematologica 2005 90 949 968 15996933

[B26] Van Etten R Shannon K Focus on myeloproliferative diseases and myelodysplastic syndromes Cancer Cell 2004 6 547 552 10.1016/j.ccr.2004.12.004 15607959

[B27] Steer E Cross N Myeloproliferative disorders with translocations of chromosome 5q31-35: role of the platelet-derived growth factor receptor Beta Acta Haematol 2002 107 113 122 10.1159/000046641 11919393

[B28] Eisen MB Spellman PT Brown PO Botstein D Cluster analysis and display of genome-wide expression patterns PNAS 1998 95 25 14863 14868 10.1073/pnas.95.25.14863 9843981 PMC24541

[B29] Deininger M Buchdunger E Druker BJ The development of imatinib as a therapeutic agent for chronic myeloid leukemia Blood 2005 105 7 2640 2653 10.1182/blood-2004-08-3097 15618470

[B30] Druker BJ Translation of the Philadelphia chromosome into therapy for CML Blood 2008 112 13 4808 4817 10.1182/blood-2008-07-077958 19064740

[B31] Salgia R Brunkhorst B Pisick E Li J Lo S Chen L Griffin J Increased tyrosine phosphorylation of focal adhesion proteins in myeloid cell lines expressing p210BCR/ABL Oncogene 1995 11 1149 1144 7566975

[B32] Nowicki M Pawlowski P Fischer T Hess G Pawlowski T Skorski T Chronic myelogenous leukemia molecular signature Oncogene 2003 22 3952 3963 10.1038/sj.onc.1206620 12813469

[B33] Der SD Zhou A Williams BRG Silverman RH Identification of genes differentially regulated by interferon alpha, beta, or gamma using oligonucleotide arrays PNAS 1998 95 26 15623 15628 10.1073/pnas.95.26.15623 9861020 PMC28094

[B34] Ohmine K Ota J Ueda M Ueno S Yoshida K Yamashita Y Kirito K Imagawa S Nakamura Y Saito K Characterization of stage progression in chronic myeloid leukemia by DNA microarray with purified hematopoietic stem cells Oncogene 2001 20 8249 8257 10.1038/sj.onc.1205029 11781839

[B35] Kronenwett R Butterweck U Steidl U Kliszewski S Neumann F Bork S Blanco E Roes N Graf T Brors B Distinct molecular phenotype of malignant CD34(+) hematopoietic stem and progenitor cells in chronic myelogenous leukemia Oncogene 2005 24 5313 5324 10.1038/sj.onc.1208596 15806158

[B36] Advani A Dressman H Quiroz M Taylor G Pendergast A Elevated expression of a subset of interferon inducible genes in primary bone marrow cells expressing p185 Bcr-Abl versus p210 Bcr-Abl by DNA microarray analysis Leuk Res 2004 28 285 294 10.1016/S0145-2126(03)00264-9 14687624

[B37] Yong ASM Szydlo RM Goldman JM Apperley JF Melo JV Molecular profiling of CD34+ cells identifies low expression of CD7, along with high expression of proteinase 3 or elastase, as predictors of longer survival in patients with CML Blood 2006 107 1 205 212 10.1182/blood-2005-05-2155 16144796

[B38] Bruns I Czibere A Fischer JC Roels F Cadeddu RP Buest S Bruennert D Huenerlituerkoglu AN Stoecklein NH Singh R The hematopoietic stem cell in chronic phase CML is characterized by a transcriptional profile resembling normal myeloid progenitor cells and reflecting loss of quiescence Leukemia 2009 23 5 892 899 10.1038/leu.2008.392 19158832

[B39] Vianello F Villanova F Tisato V Lymperi S Ho KK Gomes AR Marin D Bonnet D Apperley J Lam EW Bone marrow mesenchymal stromal cells non-selectively protect chronic myeloid leukemia cells from imatinib-induced apoptosis via the CXCR4/CXCL12 axis Haematologica 2010 95 7 1081 1089 10.3324/haematol.2009.017178 20179085 PMC2895031

[B40] Nardi V Naveiras O Azam M Daley GQ ICSBP-mediated immune protection against BCR-ABL-induced leukemia requires the CCL6 and CCL9 chemokines Blood 2009 113 16 3813 3820 10.1182/blood-2008-07-167189 19171873 PMC2670796

[B41] Eaves C Cashman J Wolpe S Eaves A Unresponsiveness of primitive chronic myeloid leukemia cells to macrophage inflammatory protein 1 alpha, an inhibitor of primitive normal hematopoietic cells PNAS 1993 90 12015 12019 10.1073/pnas.90.24.12015 8265663 PMC48116

[B42] Wark G Heyworth C Spooncer E Czaplewski L Francis J Dexter T Whetton A Abl protein kinase abrogates the response of multipotent haemopoietic cells to the growth inhibitor macrophage inflammatory protein-1 alpha Oncogene 1998 16 1319 1324 10.1038/sj.onc.1201914 9546433

[B43] Salgia R Quackenbush E Lin J Souchkova N Sattler M Ewaniuk DS Klucher KM Daley GQ Kraeft SK Sackstein R The BCR/ABL oncogene alters the chemotactic response to stromal-derived factor-1alpha Blood 1999 94 12 4233 4246 10590068

[B44] Chen YY Malik M Tomkowicz BE Collman RG Ptasznik A BCR-ABL1 alters SDF-1alpha-mediated adhesive responses through the beta2 integrin LFA-1 in leukemia cells Blood 2008 111 10 5182 5186 10.1182/blood-2007-10-117705 18339898 PMC2384141

[B45] Ptasznik A Urbanowska E Chinta S Costa MA Katz BA Stanislaus MA Demir G Linnekin D Pan ZK Gewirtz AM Crosstalk Between BCR/ABL Oncoprotein and CXCR4 Signaling through a Src Family Kinase in Human Leukemia Cells J Exp Med 2002 196 5 667 678 10.1084/jem.20020519 12208881 PMC2193994

[B46] Arm J Gurish M Reynolds D Scott H Gartner C Austen K Katz H Molecular cloning of gp49, a cell-surface antigen that is preferentially expressed by mouse mast cell progenitors and is a new member of the immunoglobulin superfamily J Biol Chem 1991 266 24 15966 15973 1714901

[B47] Daheshia M Friend DS Grusby MJ Austen KF Katz HR Increased Severity of Local and Systemic Anaphylactic Reactions in gp49B1-deficient Mice J Exp Med 2001 194 2 227 234 10.1084/jem.194.2.227 11457897 PMC2193448

[B48] Katz HR Vivier E Castells MC McCormick MJ Chambers JM Austen KF Mouse mast cell gp49B1 contains two immunoreceptor tyrosine-based inhibition motifs and suppresses mast cell activation when coligated with the high-affinity Fc receptor for†IgE PNAS 1996 93 20 10809 10814 10.1073/pnas.93.20.10809 8855262 PMC38237

[B49] Iida S Kohro T Kodama T Nagata S Fukunaga R Identification of CCR2, flotillin, and gp49B genes as new G-CSF targets during neutrophilic differentiation J Leukoc Biol 2005 78 2 481 490 10.1189/jlb.0904515 15894583

[B50] Heino J The collagen receptor integrins have distinct ligand recognition and signaling functions Matrix Biol 2000 19 319 323 10.1016/S0945-053X(00)00076-7 10963992

[B51] Gordon M Dowding C Riley G Goldman J Greaves M Altered adhesive interactions with marrow stroma of haematopoietic progenitor cells in chronic myeloid leukaemia Nature 1987 328 342 344 10.1038/328342a0 3474529

[B52] Bhatia R Wayner E McGlave P Verfaillie C Interferon-alpha restores normal adhesion of chronic myelogenous leukemia hematopoietic progenitors to bone marrow stroma by correcting impaired beta 1 integrin receptor function J Clin Invest 1994 94 384 391 10.1172/JCI117333 7518835 PMC296320

[B53] Bhatia R Verfaillie CM Inhibition of BCR-ABL expression with antisense oligodeoxynucleotides restores beta 1 integrin-mediated adhesion and proliferation inhibition in chronic myelogenous leukemia hematopoietic progenitors Blood 1998 91 9 3414 3422 9558400

[B54] Verfaillie C McCarthy J McGlave P Mechanisms underlying abnormal trafficking of malignant progenitors in chronic myelogenous leukemia. Decreased adhesion to stroma and fibronectin but increased adhesion to the basement membrane components laminin and collagen type IV J Clin Invest 1992 90 1232 1241 10.1172/JCI115985 1383271 PMC443164

[B55] Verfaillie C Hurley R Lundell B Zhao C Bhatia R Integrin-mediated regulation of hematopoiesis: do BCR/ABL-induced defects in integrin function underlie the abnormal circulation and proliferation of CML progenitors? Acta Haematol 1997 97 40 52 10.1159/000203658 8980609

[B56] Chan J Watt S Adhesion receptors on haematopoietic progenitor cells Brit J Haematol 2001 112 541 557 10.1046/j.1365-2141.2001.02439.x 11260052

[B57] Evens AM Sehn LH Farinha P Nelson BP Raji A Lu Y Brakman A Parimi V Winter JN Schumacker PT Hypoxia-inducible factor-1 {alpha} expression predicts superior survival in patients with diffuse large B-cell lymphoma treated with R-CHOP J Clin Oncol 2010 28 6 1017 1024 10.1200/JCO.2009.24.1893 20048181 PMC2834428

[B58] Wisniewski D Strife A Swendeman S Erdjument-Bromage H Geromanos S Kavanaugh WM Tempst P Clarkson B A novel SH2-containing phosphatidylinositol 3,4,5-trisphosphate 5-phosphatase (SHIP2) is constitutively tyrosine phosphorylated and associated with src homologous and collagen gene (SHC) in chronic myelogenous leukemia progenitor cells Blood 1999 93 8 2707 2720 10194451

[B59] Liang X Hajivandi M Veach D Wisniewski D Clarkson B Resh MD Pope RM Quantification of change in phosphorylation of BCR-ABL kinase and its substrates in response to Imatinib treatment in human chronic myelogenous leukemia cells Proteomics 2006 6 16 4554 4564 10.1002/pmic.200600109 16858728

[B60] Master Z Tran J Bishnoi A Chen SH Ebos JM Van Slyke P Kerbel RS Dumont DJ Dok-R binds c-Abl and regulates Abl kinase activity and mediates cytoskeletal reorganization J Biol Chem 2003 278 32 30170 30179 10.1074/jbc.M301339200 12777393

[B61] Yasuda T Shirakata M Iwama A Ishii A Ebihara Y Osawa M Honda K Shinohara H Sudo K Tsuji K Role of Dok-1 and Dok-2 in Myeloid Homeostasis and Suppression of Leukemia J Exp Med 2004 200 12 1681 1687 10.1084/jem.20041247 15611294 PMC2211997

[B62] Yasuda M Theodorakis P Subramanian T Chinnadurai G Adenovirus E1B-19 K/BCL-2 Interacting Protein BNIP3 Contains a BH3 Domain and a Mitochondrial Targeting Sequence 10.1074/jbc.273.20.12415 J Biol Chem 1998 273 20 12415 12421 10.1074/jbc.273.20.12415 9575197

[B63] Rodriguez del Castillo A Vitale M Tchakarov L Trifaró J Human platelets contain scinderin, a Ca2+-dependent actin filament-severing protein Thromb Haemost 1991 67 248 251 1621245

[B64] Unwin RD Sternberg DW Lu Y Pierce A Gilliland DG Whetton AD Global effects of BCR/ABL and TEL/PDGFR-beta expression on the proteome and phosphoproteome: identification of the rho pathway as a target of BCR/ABL J BiolChem 2005 280 8 6316 6326 10.1074/jbc.M410598200 15569670

[B65] Zunino R Li Q Rose SD Romero-Benitez MMI Lejen T Brandan NC Trifaro J-M Expression of scinderin in megakaryoblastic leukemia cells induces differentiation, maturation, and apoptosis with release of plateletlike particles and inhibits proliferation and tumorigenesis Blood 2001 98 7 2210 2219 10.1182/blood.V98.7.2210 11568009

[B66] Tam WF Gu TL Chen J Lee BH Bullinger L Frohling S Wang A Monti S Golub TR Gilliland DG Id1 is a common downstream target of oncogenic tyrosine kinases in leukemic cells Blood 2008 112 5 1981 1992 10.1182/blood-2007-07-103010 18559972 PMC2518899

[B67] Perk J Iavarone A Benezra R Id family of helix-loop-helix proteins in cancer Nat Rev Cancer 2005 5 603 614 10.1038/nrc1673 16034366

[B68] Xu M Nie L Kim S Sun X STAT5-induced Id-1 transcription involves recruitment of HDAC1 and deacetylation of C/EBPbeta EMBO J 2003 22 893 904 10.1093/emboj/cdg094 12574125 PMC145454

[B69] Cain JA Xiang Z O'Neal J Kreisel F Colson A Luo H Hennighausen L Tomasson MH Myeloproliferative disease induced by TEL-PDGFRB displays dynamic range sensitivity to Stat5 gene dosage Blood 2007 109 9 3906 3914 10.1182/blood-2006-07-036335 17218386 PMC1874559

