# Supplementary material for: Tyrosine kinase chromosomal translocations mediate distinct and overlapping gene regulation events
Source: BMC Cancer. 2011 Dec 28;11:528. doi: 10.1186/1471-2407-11-528 (PMC3295743; doi:10.1186/1471-2407-11-528)
Supplement: Additional file 1 — Table S1. Primer sequences used for Q-PCR. [file 1471-2407-11-528-S1.DOC]

**SUPPLEMENTARY TABLE 1**

**Primer Sequences Used for Q-PCR**

Col5a1 Forward CCCTCCGTGGCATTGGA

Col5a1 Reverse GACACGGCTCTTTCCCTTTG

Cxcl10 Forward GCCATGGTCCTGAGACAAAAG

Cxcl10 Reverse GACTCAGACCAGCCCTTAAAGAAT

Cyclophilin H Forward AGCTGGACTGGCCTCTGTGT

Cyclophilin H Reverse CAGGTCCTGGATCGCATACTG

Daf1 Forward GAAATGAAGAATGTTGAACCTTTTGA

Daf1 Reverse CGTGGTCTCCAACCACTTCCT

Dok2 Forward ATGGCCAGAAGATGGGATATGT

Dok2 Reverse CCATTTCCCCTCCACAGCTT

Gp49b Forward CATGCAAAGAGACCCAGGATGTA

Gp49b Reverse GTTGTTCTGTTCCTGTGTCCTGAT

Id1 Forward GAACGTCCTGCTCTACGACATG

Id1 Reverse TGGGCACCAGCTCCTTGA

Isg20 Forward TCAATGCCCTGAAGGAGGAT

Isg20 Reverse GCCTGTCTGTGGACGTGTCA

Mrvi1 Forward GGGATGGCATGGCTGAAA

Mrvi1 Reverse CTGTGACTCGCCTTCCTGAAC

Nrdg1 Forward AATGCCGGGCCCAAGT

Nrdg1 Reverse GGTGACTGATGCACAGATTGCT

Scinderin Forward CCCCGTCTGCTACTCTGTTTG

Scinderin Reverse GGAAACAGCAAGGCATTGTTAAA

Stat1 Forward TTGTGGCACGTGTGTGATCA

Stat1 Reverse GCTGGCCCAGAAGAACCAA
